# Supplementary material for: SpinCastML—An Open Decision-Making Application for Inverse Design of Electrospinning Manufacturing: A Machine Learning, Optimal Sampling, and Inverse Monte Carlo Approach
Source: ACS Omega. 2026 Aug 12;11(33):50531–50. doi: 10.1021/acsomega.6c07333 (PMC13520440; doi:10.1021/acsomega.6c07333)
Supplement: Supplementary file 1 [file ao6c07333_si_001.pdf]

SpinCastML an Open Decision-Making Application for Inverse Design of Electrospinning Manufacturing: A Machine Learning, Optimal Sampling and Inverse Monte Carlo Approach

Elisa Roldán[1]*, Tasneem Sabir[2]

[1] Department of Engineering, Faculty of Science & Engineering, Manchester Metropolitan University, Manchester M1 5GD, UK

[2] Manchester Fashion Institute, Faculty of Arts & Humanities, Manchester Metropolitan University, Manchester M15 6BG, UK

*** Correspondence:**
Elisa.Roldan-Ciudad@mmu.ac.uk

Table S1 Supplementary. Descriptive analysis of electrospun fiber diameters

	Statistics			Polymer															
				CA	GELATIN	Nylon-6	PAN	PCL	PDLLA	PEEK-sul	PET	PLA	PMMA	PS	PU	PVA	PVDF	PVP	Y_PGA
Fiber diameter (nm)	Mean		Estadístico	538.9	368.4	372.6	210.5	356.7	840.0	187.8	404.0	378.2	614.2	987.7	372.9	264.8	689.4	1102.9	315.4
			Standard error	8.4	7.5	6.5	0.9	3.7	31.2	0.6	7.9	9.0	7.3	22.2	3.9	1.6	5.2	26.0	3.6
	95% confidence interval	Inferior limit	Estadístico	522.3	353.6	359.8	208.7	349.4	778.7	186.5	388.5	360.5	599.8	944.1	365.2	261.7	679.1	1051.9	308.3
		Superior limit	Estadístico	555.4	383.2	385.4	212.2	364.0	901.2	189.1	419.6	395.9	628.6	1031.3	380.6	268.0	699.6	1153.8	322.5
	Median		Estadístico	488.2	258.6	209.0	201.7	324.8	633.2	183.6	399.0	308.4	430.4	549.7	381.2	246.0	444.5	554.8	293.6
	Variance		Estadístico	133804.9	95428.7	67900.2	3544.9	23838.6	622397.0	479.6	30079.5	26016.5	256902.8	888222.3	10484.0	18063.0	959893.2	3296020.4	9525.5
	Standard deviation		Estadístico	365.8	308.9	260.6	59.5	154.4	788.9	21.9	173.4	161.3	506.9	942.5	102.4	134.4	979.7	1815.5	97.6
	Minimum		Estadístico	125.1	39.9	77.4	90.5	113.1	79.8	145.7	181.7	199.3	95.1	163.1	183.7	40.0	25.0	77.0	172.9
	Maximum		Estadístico	1515.6	1990.0	831.8	453.0	824.9	2586.3	259.5	763.3	725.4	2571.7	4326.6	572.6	995.1	8706.9	14874.9	630.9
	Range		Estadístico	1390.5	1950.1	754.4	362.4	711.8	2506.6	113.8	581.7	526.1	2476.6	4163.5	389.0	955.1	8681.9	14798.0	458.0
	Interquartile range (IQR)		Estadístico	634.5	398.1	510.7	69.3	120.8	1149.4	29.2	317.6	284.0	390.6	1039.5	104.9	92.5	512.3	850.1	98.2
	Skewness		Estadístico	0.50	1.67	0.47	0.79	0.98	1.03	0.72	0.30	0.75	1.60	1.78	-0.27	1.86	4.51	4.09	1.38
			Standard error	0.06	0.06	0.06	0.04	0.06	0.10	0.07	0.11	0.14	0.04	0.06	0.09	0.03	0.01	0.04	0.09
	Kurtosis		Estadístico	-0.79	3.29	-1.63	1.02	0.46	-0.07	-0.06	-1.06	-0.83	2.06	2.93	-0.80	4.71	24.81	19.26	1.91
			Standard error	0.11	0.12	0.12	0.07	0.12	0.19	0.14	0.22	0.27	0.07	0.12	0.19	0.06	0.03	0.07	0.18

Table S2 Supplementary. Impact on sample size (random sampling)

Sample size	Computational time	Set	RMSE	MAE	MAPE	R^2
60000	11'02''	CV(train)	254.253	69.673	14.191	0.925
		Test(30%)	231.965	65.310	13.633	0.933
40000	7'29''	CV(train)	266.357	72.021	14.666	0.919
		Test(30%)	266.654	71.943	14.766	0.918
20000	3'48''	CV(train)	233.353	70.009	15.074	0.935
		Test(30%)	268.708	71.697	15.377	0.921
10000	2'01''	CV(train)	214.581	57.640	10.221	0.931
		Test(30%)	284.550	56.403	8.641	0.939
8000	1'36''	CV(train)	338.105	80.113	16.686	0.862
		Test(30%)	181.507	69.137	15.861	0.957
6000	1'21''	CV(train)	185.093	71.514	16.314	0.952
		Test(30%)	338.333	80.638	17.055	0.876

4000	59''	CV(train)	242.648	82.316	17.565	0.926
		Test(30%)	338.240	87.045	17.314	0.880
2000	42''	CV(train)	334.741	126.622	28.885	0.854
		Test(30%)	566.055	136.738	25.895	0.759

Table S3 Supplementary. Performance of the Inverse Monte Carlo (IMC) framework across polymers, sampling strategies and IMC modes

Sampling	Polymer	Mode	Pred_mean	Pred_sd	Obs_mean	Obs_sd	Success model	Success Observed
Random	CA	Experimental	504.06	351.59	509.25	359.94	40.60	37.60
Random	CA	Optimized	509.31	355.51	509.25	359.94	39.80	37.60
Random	PAN	Experimental	208.48	58.23	208.88	58.19	63.60	62.60
Random	PAN	Optimized	361.18	366.92	208.88	58.19	31.80	62.60
Random	PCL	Experimental	360.53	157.13	362.35	161.23	62.10	63.70
Random	PCL	Optimized	480.95	232.54	362.35	161.23	55.60	63.70
Random	PDLLA	Experimental	864.64	771.84	866.10	808.51	86.70	86.00
Random	PDLLA	Optimized	1111.56	798.71	866.10	808.51	86.50	86.00
Random	PET	Experimental	406.20	185.54	404.72	189.82	54.70	53.40
Random	PET	Optimized	1163.72	1562.31	404.72	189.82	55.30	53.40
Random	PLA	Experimental	389.85	150.22	390.81	155.12	67.70	63.30
Random	PLA	Optimized	405.54	110.66	390.81	155.12	87.10	63.30
Random	PS	Experimental	988.20	880.49	987.27	945.82	87.40	87.50
Random	PS	Optimized	711.74	389.50	987.27	945.82	98.80	87.50
Random	PVA	Experimental	258.21	117.73	258.85	125.72	72.50	75.40
Random	PVA	Optimized	630.17	425.89	258.85	125.72	29.10	75.40
Random	PVDF	Experimental	670.62	935.16	668.83	935.05	92.60	92.50
Random	PVDF	Optimized	1453.56	1059.20	668.83	935.05	63.40	92.50
Random	PVP	Experimental	1145.40	1958.76	1126.53	1992.37	93.20	94.10
Random	PVP	Optimized	1666.11	1279.28	1126.53	1992.37	88.00	94.10
Random	Y-PGA	Experimental	321.76	49.70	326.06	109.96	100.00	79.60
Random	Y-PGA	Optimized	457.63	194.41	326.06	109.96	52.90	79.60
Random	PMMA	Experimental	600.43	501.47	598.66	507.94	81.70	82.20
Random	PMMA	Optimized	1022.13	882.69	598.66	507.94	68.90	82.20
Random	GELATIN	Experimental	357.46	283.99	361.42	305.25	86.10	82.00
Random	GELATIN	Optimized	998.53	700.41	361.42	305.25	47.00	82.00
Random	PEEK-sulfonated	Experimental	190.55	12.39	187.96	20.96	91.00	59.70
Random	PEEK-sulfonated	Optimized	185.76	21.78	187.96	20.96	96.80	59.70
Random	PU	Experimental	365.46	106.80	363.09	107.21	51.40	52.00
Random	PU	Optimized	408.61	180.01	363.09	107.21	49.30	52.00
Random	Nylon-6	Experimental	390.60	261.90	390.47	263.98	50.20	48.60
Random	Nylon-6	Optimized	633.26	194.57	390.47	263.98	47.00	48.60

Sobol+D-optimal	CA	Experimental	500.59	372.20	503.33	375.78	20.50	27.30
Sobol+D-optimal	CA	Optimized	882.54	758.84	503.33	375.78	39.60	27.30
Sobol+D-optimal	PAN	Experimental	213.98	60.99	212.62	60.07	75.80	73.30
Sobol+D-optimal	PAN	Optimized	447.53	599.40	212.62	60.07	34.90	73.30
Sobol+D-optimal	PCL	Experimental	360.15	164.96	358.42	165.71	66.90	61.40
Sobol+D-optimal	PCL	Optimized	429.49	204.52	358.42	165.71	58.40	61.40
Sobol+D-optimal	PDLLA	Experimental	802.93	703.49	806.73	739.74	81.70	74.70
Sobol+D-optimal	PDLLA	Optimized	1080.98	622.91	806.73	739.74	72.60	74.70
Sobol+D-optimal	PET	Experimental	394.79	165.35	400.13	173.95	48.70	46.80
Sobol+D-optimal	PET	Optimized	1007.00	1280.09	400.13	173.95	40.50	46.80
Sobol+D-optimal	PLA	Experimental	379.93	146.94	374.95	150.99	66.80	55.80
Sobol+D-optimal	PLA	Optimized	298.99	112.80	374.95	150.99	66.40	55.80
Sobol+D-optimal	PS	Experimental	984.57	918.51	1005.09	977.81	94.90	87.00
Sobol+D-optimal	PS	Optimized	742.43	391.62	1005.09	977.81	99.90	87.00
Sobol+D-optimal	PVA	Experimental	264.74	132.77	265.39	134.76	75.60	78.20
Sobol+D-optimal	PVA	Optimized	580.99	366.43	265.39	134.76	35.60	78.20
Sobol+D-optimal	PVDF	Experimental	671.12	938.59	668.65	938.53	92.90	92.90
Sobol+D-optimal	PVDF	Optimized	1633.53	1285.04	668.65	938.53	59.80	92.90
Sobol+D-optimal	PVP	Experimental	1160.45	1691.50	1166.61	1949.17	94.40	93.50
Sobol+D-optimal	PVP	Optimized	2334.49	2057.93	1166.61	1949.17	64.60	93.50
Sobol+D-optimal	Y_PGA	Experimental	309.24	53.21	313.53	101.05	88.40	68.60
Sobol+D-optimal	Y_PGA	Optimized	467.97	171.95	313.53	101.05	42.60	68.60
Sobol+D-optimal	PMMA	Experimental	624.48	514.60	631.27	520.85	80.80	80.90
Sobol+D-optimal	PMMA	Optimized	951.31	959.11	631.27	520.85	75.30	80.90
Sobol+D-optimal	GELATIN	Experimental	355.73	256.83	359.77	289.88	90.20	86.80

Sobol+D-optimal	GELATIN	Optimized	973.58	517.68	359.77	289.88	32.20	86.80
Sobol+D-optimal	PEEK-sulfonated	Experimental	188.93	15.47	186.78	22.57	81.50	58.30
Sobol+D-optimal	PEEK-sulfonated	Optimized	179.78	11.03	186.78	22.57	81.60	58.30
Sobol+D-optimal	PU	Experimental	367.92	105.76	366.71	110.56	55.60	54.60
Sobol+D-optimal	PU	Optimized	421.49	143.38	366.71	110.56	57.30	54.60
Sobol+D-optimal	Nylon-6	Experimental	371.36	261.84	375.53	263.51	53.50	52.60
Sobol+D-optimal	Nylon-6	Optimized	661.23	269.69	375.53	263.51	61.10	52.60
Balanced	CA	Experimental	555.56	393.83	532.16	387.72	76.10	75.10
Balanced	CA	Optimized	1227.60	826.10	532.16	387.72	47.10	75.10
Balanced	PAN	Experimental	211.83	59.95	211.80	62.72	69.30	69.70
Balanced	PAN	Optimized	367.70	172.24	211.80	62.72	25.50	69.70
Balanced	PCL	Experimental	377.62	163.10	374.79	163.90	65.50	64.00
Balanced	PCL	Optimized	595.25	229.48	374.79	163.90	38.90	64.00
Balanced	PDLLA	Experimental	952.67	846.71	948.22	861.70	83.00	79.80
Balanced	PDLLA	Optimized	1326.36	918.91	948.22	861.70	73.50	79.80
Balanced	PET	Experimental	438.55	165.73	436.85	172.03	53.40	56.10
Balanced	PET	Optimized	1086.29	1244.67	436.85	172.03	45.00	56.10
Balanced	PLA	Experimental	351.27	137.18	350.98	143.29	77.80	79.50
Balanced	PLA	Optimized	430.99	204.86	350.98	143.29	35.60	79.50
Balanced	PS	Experimental	1054.85	978.29	1033.92	994.09	82.70	86.50
Balanced	PS	Optimized	736.89	397.54	1033.92	994.09	99.20	86.50
Balanced	PVA	Experimental	258.54	120.35	257.56	127.06	74.70	76.80
Balanced	PVA	Optimized	621.64	293.00	257.56	127.06	19.50	76.80
Balanced	PVDF	Experimental	689.19	957.46	690.10	969.62	92.50	93.20
Balanced	PVDF	Optimized	1588.39	1192.72	690.10	969.62	63.80	93.20
Balanced	PVP	Experimental	1056.97	1657.08	1070.00	1905.15	87.60	88.30
Balanced	PVP	Optimized	1776.89	1760.20	1070.00	1905.15	64.20	88.30
Balanced	Y_PGA	Experimental	308.43	40.90	315.45	100.70	97.90	77.30
Balanced	Y_PGA	Optimized	579.42	261.36	315.45	100.70	38.90	77.30
Balanced	PMMA	Experimental	620.89	526.94	606.14	521.31	80.80	82.20
Balanced	PMMA	Optimized	1154.82	949.74	606.14	521.31	65.10	82.20
Balanced	GELATIN	Experimental	357.92	278.42	363.66	298.73	84.40	83.50
Balanced	GELATIN	Optimized	913.51	519.44	363.66	298.73	39.90	83.50
Balanced	PEEK-sulfonated	Experimental	188.85	14.60	188.61	22.05	85.50	58.80
Balanced	PEEK-sulfonated	Optimized	258.91	25.35	188.61	22.05	0.00	58.80
Balanced	PU	Experimental	387.84	90.23	386.99	97.47	64.40	64.40
Balanced	PU	Optimized	524.11	321.36	386.99	97.47	24.10	64.40
Balanced	Nylon-6	Experimental	364.22	252.45	366.74	253.58	54.60	54.30

Balanced	Nylon-6	Optimized	557,18	270,65	366,74	253,58	74,2	54.30

Table S4 Supplementary. Machine Learning Models Metrics for k = 3 and 10

Model	Set	CV - 3				CV - 10			
		RMSE	MAE	MAPE	R2	RMSE	MAE	MAPE	R2
RF	CV (train),	515.34	183.89	44.97	0.714352672	511.23	182.00	44.64	0.72103436
RF	Test (30%)	530.82	183.89	43.34	0.719923871	537.23	184.11	43.68	0.70909410
Ranger	CV (train)	514.27	184.68	45.29	0.716378933	511.89	181.96	44.85	0.72023967
Ranger	Test (30%)	530.97	184.05	43.42	0.720418097	543.93	186.20	44.14	0.70034135
svmRadial	CV (train)	690.19	203.52	35.95	0.460333217	673.86	193.87	34.64	0.48142370
svmRadial	Test (30%)	700.88	194.38	34.15	0.464003567	699.19	193.68	34.06	0.46609036
glmnet	CV (train)	686.82	296.60	75.02	0.41925418	685.34	296.57	75.32	0.42172124
glmnet	Test (30%)	705.60	294.79	72.54	0.421192617	705.62	294.38	72.42	0.42117305
rpart	CV (train)	591.85	336.19	109.59	0.568816864	603.40	339.30	109.77	0.55216490
rpart	Test (30%)	617.60	346.01	111.00	0.557322367	617.60	346.01	111.00	0.55732237
Lm	CV (train)	691.67	298.30	75.64	0.411208087	686.42	297.05	75.54	0.41998203
Lm	Test (30%)	705.52	295.17	72.73	0.42132176	705.52	295.17	72.73	0.42132176
KNN	CV (train)	423.39	106.31	19.54	0.779424234	379.73	89.92	16.07	0.82281769
KNN	Test (30%)	318.88	80.32	15.50	0.881795189	318.88	80.32	15.50	0.88179519
xgbTree	CV (train)	**264.98**	**79.06**	**17.16**	**0.913700741**	**284.20**	**76.62**	**16.52**	**0.90067130**
xgbTree	Test (30%)	**264.89**	**75.73**	**16.30**	**0.920066691**	**264.75**	**74.93**	**16.13**	**0.91987570**
Tierra	CV (train)	598.25	290.60	83.57	0.559951278	599.78	292.94	85.33	0.55797537
Tierra	Test (30%)	599.04	292.08	87.02	0.582868845	599.04	292.08	87.02	0.58286885
Cubista	CV (train)	**277.52**	**62.15**	**10.04**	**0.905351648**	**270.58**	**60.03**	**9.47**	**0.90987201**
Cubista	Test (30%)	**261.65**	**55.58**	**8.09**	**0.921356714**	**265.01**	**59.30**	**8.99**	**0.91917260**
GBM	CV (train)	295.24	124.78	31.95	0.892737515	318.12	125.34	32.13	0.87540983
GBM	Test (30%)	299.85	127.92	32.88	0.896961564	296.95	126.13	33.67	0.89885650

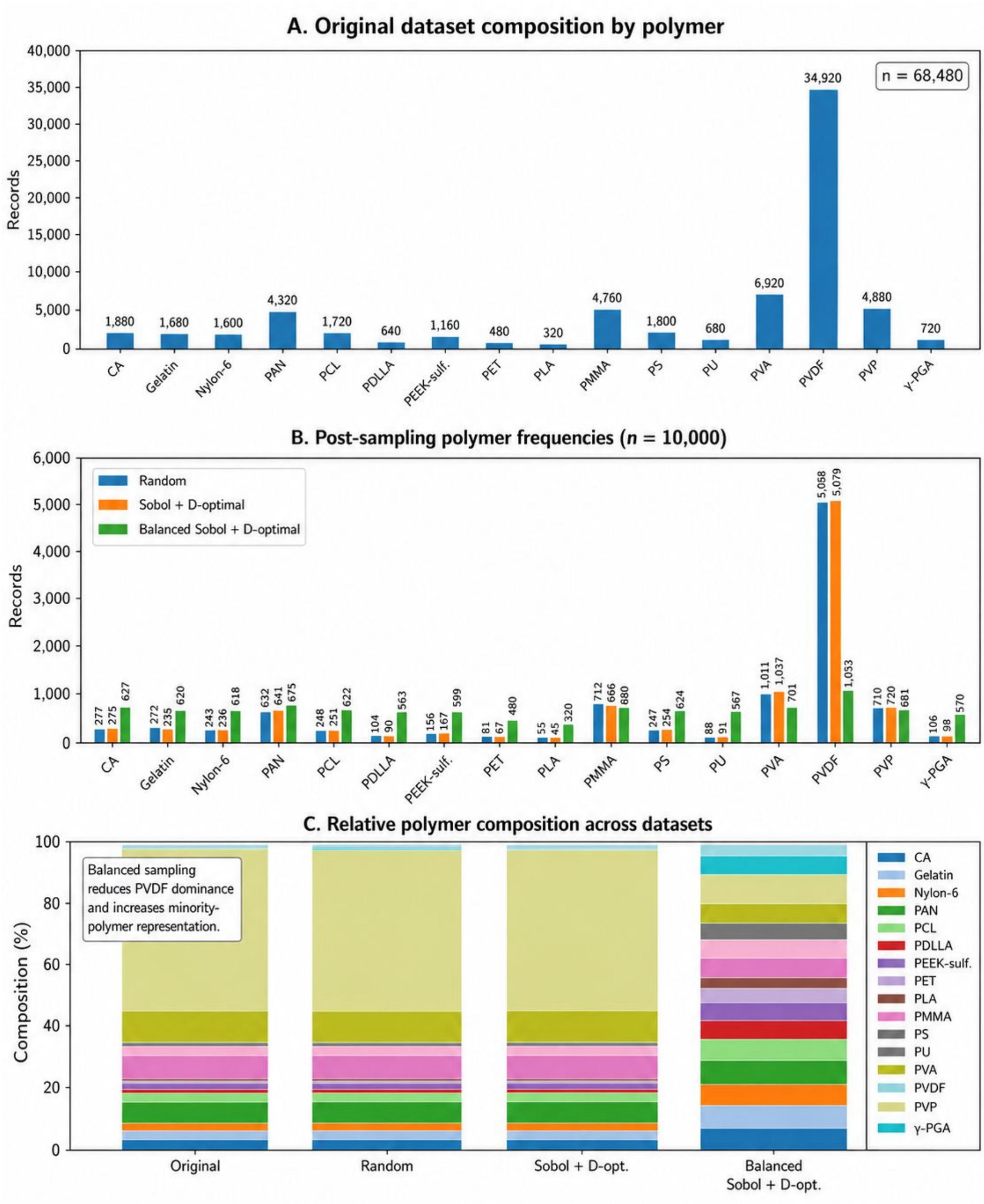

Figure S1 Supplementary. Polymer distribution before and after sampling. (A) Original dataset composition across 16 polymers. (B) Polymer frequencies after random, Sobol+D-optimal, and polymer-balanced Sobol+D-optimal sampling. (C) Relative polymer composition across datasets.

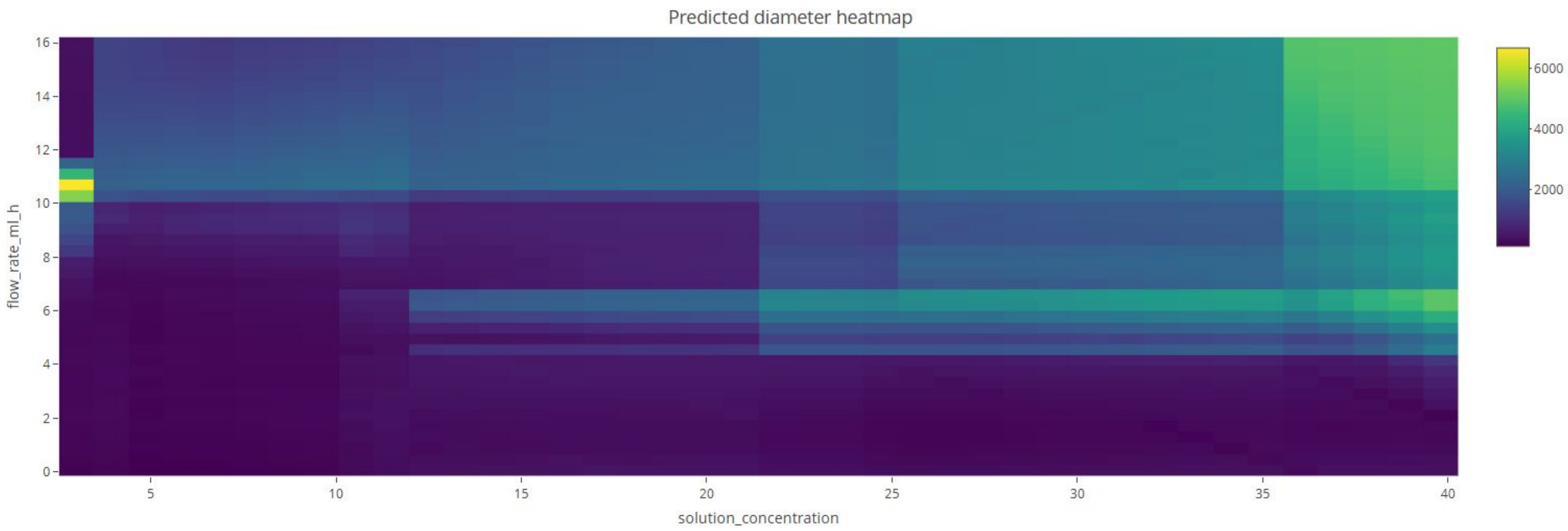

Figure S2 Supplementary. Predicted diameter heatmap

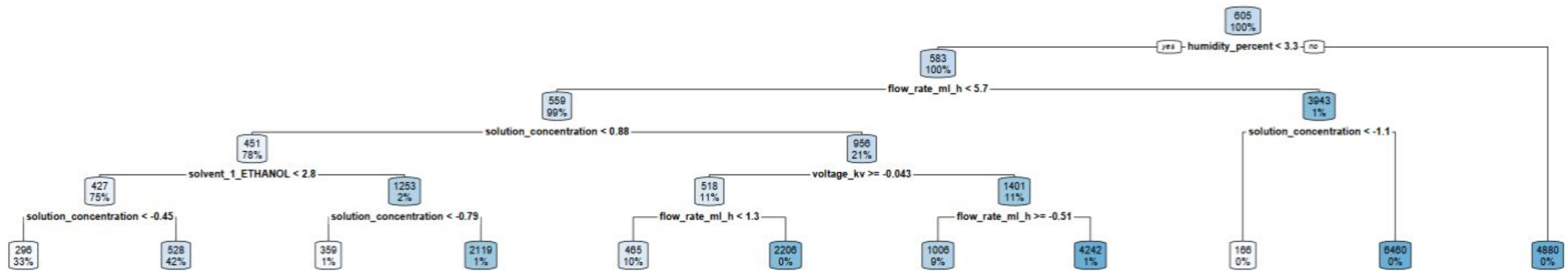

Figure S3 Supplementary. Decision Tree (rpart)

Table S5 Supplementary. 20 chemically feasible PVA formulation–process families from the prospective case study (selected parameters highlighted in yellow)

polymer	collector type	solvent 1	solvent 1 ratio	solvent 2	solvent 2 ratio	solvent 3	solvent 3 ratio	solution concentration	needle diameter (g)	rotation speed (rpm)	voltage(kv)	flow rate (ml/h)	tip collector distance (cm)	temperature (ºC)	humidity _percent	solubility flag	pred	error	error abs
PVA	Flat	WATER	100		0		0	8	22	0	20	0.4	10	20	45	OK	249.53	-0.47	0.47
PVA	Flat	WATER	100		0		0	8	22	0	15	0.4	10	20	45	OK	251.877	1.877	1.877
PVA	Flat	WATER	100		0		0	10	22	0	15	0.4	20	20	45	OK	252.909	2.909	2.909
PVA	Flat	WATER	100		0		0	9	22	0	22.5	0.25	20	20	45	OK	252.992	2.992	2.992
PVA	Flat	WATER	100		0		0	10	22	0	15	0.2	20	23	45	OK	253.19	3.19	3.19
PVA	Flat	WATER	100		0		0	12	22	0	25	0.3	10	20	45	OK	246.189	-3.811	3.811
PVA	Flat	WATER	100		0		0	8	22	0	15	0.4	15	20	45	OK	246.106	-3.894	3.894
PVA	Flat	WATER	100		0		0	12	22	0	25	0.4	10	20	45	OK	254.561	4.561	4.561
PVA	Flat	WATER	100		0		0	10	22	0	15	0.25	12.5	20	45	OK	255.672	5.672	5.672
PVA	Flat	WATER	100		0		0	7.4	9	0	13	0.2	15	23	45	OK	243.627	-6.373	6.373
PVA	Flat	WATER	100		0		0	10	22	0	25	0.4	10	20	45	OK	243.564	-6.436	6.436
PVA	Flat	WATER	100		0		0	9	22	0	15	0.3	12.5	20	45	OK	243.269	-6.731	6.731
PVA	Flat	WATER	100		0		0	10	22	0	22.5	0.25	12.5	20	45	OK	256.995	6.995	6.995
PVA	Flat	WATER	100		0		0	10	22	0	15	0.2	15	20	45	OK	257.594	7.594	7.594
PVA	Flat	WATER	100		0		0	10	22	0	20	0.2	10	20	45	OK	257.676	7.676	7.676
PVA	Flat	WATER	100		0		0	10	22	0	15	0.3	20	20	45	OK	241.894	-8.106	8.106
PVA	Flat	WATER	100		0		0	10	22	0	22.5	0.3	12.5	20	45	OK	258.897	8.897	8.897
PVA	Flat	WATER	100		0		0	8	22	0	15	0.3	10	20	45	OK	240.569	-9.431	9.431
PVA	Flat	WATER	100		0		0	9	22	0	15	0.25	12.5	20	45	OK	240.274	-9.726	9.726
PVA	Flat	WATER	100		0		0	10	22	0	15	0.3	12.5	20	45	OK	259.73	9.73	9.73
